# Suicide related events and attention deficit hyperactivity disorder treatments in children and adolescents: a meta-analysis of atomoxetine and methylphenidate comparator clinical trials

**DOI:** 10.1186/1753-2000-7-19

**Published:** 2013-06-19

**Authors:** Chris J Bushe, Nicola C Savill

**Affiliations:** 1 Eli Lilly and Company Ltd, Lilly House, Priestley Road, Basingstoke RG24 9NL, United Kingdom

**Keywords:** ADHD, Suicide-related events, Summary of product characteristics, Systematic review, Atomoxetine, Methylphenidate

## Abstract

**Background:**

Attention Deficit Hyperactivity Disorder (ADHD) is becoming an increasingly commonly diagnosed and treated childhood illness. Untreated ADHD is recognised as an independent risk factor for suicide-related events and deliberate self-harm and is reported more commonly in these populations. With the treatment of ADHD it is thus crucial to understand further any associations between pharmacological treatments and suicide-related events. Specific data for suicide-related events with stimulants have not been publically reported. Suicidal tendencies are, however, a contraindication to the treatment of patients with methylphenidate. Clinicians and patients may be helped by a meta-analytic comparison of suicide-related events in comparative randomised double-blind atomoxetine and methylphenidate clinical trials.

**Methods:**

Suicide-related events retrospectively mapped to the suicide-related event assessment instrument recommended by the FDA, the Columbia Classification Algorithm for Suicide Assessment (C-CASA), were evaluated in five double-blind placebo controlled comparative studies of atomoxetine and methylphenidate (n = 1024) of 6 to 9 weeks duration. The Mantel-Haenszel risk ratio and Mantel-Haenszel incidence differences have been calculated.

**Results:**

In total there were 5 suicide-related events, atomoxetine (ATX) 3/559 and methylphenidate (MPH) 2/465. There were no suicide attempts nor completed suicides. Meta-analysis finds no difference of a difference in risk between ATX and MPH with a Mantel-Haenszel risk ratio of 0.52 (95% CI; 0.06, 4.54).

**Conclusion:**

In the only reported meta-analysis of comparative suicide-related events between atomoxetine and methylphenidate, no significant evidence of a difference in risk has been found. These data may be informative to clinicians and patients when developing clinical guidelines.

## Introduction

Atomoxetine was first licensed in Europe in 2004 and is currently the only non-stimulant medication licensed in Europe for the treatment of attention deficit hyperactivity disorder (ADHD) in children and adolescents. As of January 2012 there are six other medications in the UK also licensed, with some available in other European countries, for ADHD, of which five are methylphenidate formulations and dexamphetamine. There is good evidence that the rates of both diagnosis and treatment of ADHD have been increasing over the last decade. During 1999–2006 the prevalence of prescribing of ADHD medications in the age group 15–21 in the UK increased 6.23 fold [1]. New data from the Center for Disease Control and Prevention (CDC) using parental reports finds that ADHD prevalence has increased from 7% to 9% (children aged 5–17) when comparing 1998–2000 and 2007–2009 [2].

In a paediatric population there is a clear focus on the safety of medications and this is paramount when considering suicidality. Suicide-related events are a broad term that encompasses suicidal ideation, behaviours, attempts and completed suicides. ADHD is an illness with which comorbid depression and anxiety are commonly found and knowledge regarding the incidence of self-harm in the adolescent group is now available [3]. Recent cohort studies have reported that rates of suicidal ideation, deliberate self-harm (DSH) and suicide are significantly increased in untreated ADHD populations [4,5]. An analysis of six prospective studies measuring annual suicide rates reported a comparative risk of 2.91 for males (5–24 years) in comparison to the general population [4], suggesting that ADHD may increase the severity of comorbid conditions (conduct disorder and depression). In a study from the Northern Finland 1986 birth cohort in a treatment-naïve cohort, suicidal ideation by age 15–16 years was reported in 51% of the ADHD cohort and 24% of the non-ADHD cohort, with a clear conclusion that the illness ADHD is a risk factor for both suicidal ideation and DSH [5]. DSH was reported in 30% of the ADHD cohort compared to 8% of the non-ADHD cohort. There were no completed suicides. An Australian cohort study of 1802 general population adolescents followed up when aged 14 to 19 years reported self-harm in 8% (149/1802), of whom self-harm with suicidal intention comprised 0.8% (15/1802) [6]. The commonest form of self-harm was cutting or burning (4.6%), poison or overdose (1.9%), and risk taking (1.7%). The number of subjects with ADHD in this cohort was not stated. Anti-social behaviour, depression, and anxiety were also found to be independently associated with self-harm. These comorbid disorders are commonly found in ADHD populations [4]. These data seem in line with data showing that risk of suicidal behaviours is significant in adolescent populations [1,5,6].

The next relevant clinical question relates to the association of ADHD treatments with suicidality. In 2008 from a meta-analysis of suicide-related events from randomised clinical trials in atomoxetine patients, there was a clear conclusion that, although uncommon, suicidal ideation was significantly more common in paediatric ADHD patients receiving atomoxetine than placebo [7]. For methylphenidate and other stimulants specific figures are not publically available however the relevant summaries of product characteristics (SPC) provide advice for and mandate monitoring [4]. Clinicians can also seek independent advice on suicidality from national guidance (in the United Kingdom this would be NICE and SIGN), relevant summaries of product characteristics (SPC), European expert groups and other worldwide developed guidance [3,8-11]. Two recent systematic reviews have emphasised that there is dissonance between these information sources [10,11], especially in terms of the comparative suicide-related events data [7] relating to atomoxetine and methylphenidate. In a systematic review of review papers on atomoxetine from 2009–2011, a clear finding emerged that relevant comparative suicide-related events data available at the time of publication of the individual reviews were rarely included [10]. The comparative data are also not well reported in many clinical papers [3]. In 2011 the American Academy of Pediatrics published their clinical practice guideline for ADHD and similarly only refer to atomoxetine in relation to an increase in suicidal thoughts [12]. No mention is made of suicide-related events in relation to any psychostimulant [12].

Because suicide-related events are so rare, individual clinical trials are too small to collect data on either the incidence of such events or comparative incidence rates [13]. This is evidenced by data from the national register on ADHD from the Lombardy region of Italy where, in 130 treatment-naïve subjects followed for 1 year receiving atomoxetine or methylphenidate, there were no reported suicide-related events of any type [14]. Due to the relatively low number of suicide-related events, amalgamation of clinical trials through meta-analysis may provide clinically relevant data for clinicians.

There are a number of studies comparing atomoxetine and methylphenidate. A recent non-inferiority meta-analysis considering core ADHD symptoms included 7 direct comparative atomoxetine and methylphenidate clinical trials of at least 6 weeks duration (n = 1368) reporting no efficacy differences in responder rates [15]. Meta-analysis is thus a tool that can be effectively utilised for combining studies effectively. The aim of our meta-analysis is to combine the clinical trial database of comparative randomised double-blind trials conducted by Eli Lilly involving methylphenidate and atomoxetine that report suicide-related events that can be coded to the FDA preferred reporting terms as defined within the Columbia Classification Algorithm of Suicide Assessment (C-CASA) [16-18].

In 2010 the FDA recommended that in all trials in psychiatric populations an assessment instrument mapping to the Columbia Classification Algorithm for Suicide Assessment (C-CASA) should be routinely used [16-18]. C-CASA provides a set of 9 preferred terms to code suicide-related adverse events in clinical trials [16]. The two specific purposes are to prospectively capture not only all suicidal outcomes but, by asking simple predefined questions, to be able to code them accurately to levels of severity and suicidal intent. The assessment tool the FDA specifically cites to aid this process is the Columbia Suicide Severity Rating Scale (C-SSRS) [16-18]. At the time these comparative studies of atomoxetine and methylphenidate were conducted this rating scale was not defined and operational. However, the clinical reports make feasible a retrospective classification of all suicide-related events from the trials. This meta-analysis reports in detail the outcomes. Some data from this meta-analysis have been previously published [7].

## Methods

An analysis of suicide-related events identified in paediatric randomised controlled double-blind ADHD clinical studies involving both atomoxetine and methylphenidate undertaken by Eli Lilly (studies HFBD, HFBK, LYAV, LYBI and LYBR) was conducted [19-22]. Table 1 gives study details including exclusion criteria. All data included derives from prospectively collected data as part of a clinical trial. All studies involved treatment with the active comparator methylphenidate. Analyses were conducted using FDA-defined search methodology [16-18]. None of these trials were safety trials with a primary safety endpoint, all being powered on efficacy variables, with safety data being routinely collected. All these trials are published in peer reviewed journals.

**Table 1:** Acute, paediatric, active comparator-controlled studies in ADHD

**Study acronym**	**Study design**	**Start/Stop dates**	**Study duration (weeks)**	**Age range (years)**	**Numbers**	**Inclusion criteria**	**Exclusion criteria**
HFBD [19]	DB,MC,PC,R	Nov 1998/Feb	9	7-12	65 ATX	ADHD diagnosis, normal intelligence, minimum severity criteria	PMs, <25 kg, history of BPD I/II, psychosis/OBD/seizures,on psychotropic medication, history (3 m) of drug/alcohol abuse, significant prior or current medical conditions
(Spencer;2002)		2000			62 Pbo		
					20 MPH		
HFBK [19]	DB,MC,PC,R	Nov 1998/Feb	9	7-12	64 ATX	ADHD diagnosis, normal intelligence, minimum severity criteria	PMs,<25 kg, history of BPD I/II, psychosis/OBD/seizures, on psychotropic medication, history (3 m) of drug/alcohol abuse, significant prior or current medical conditions
(Spencer;2002)		2000			62 Pbo		
					18 MPH		
LYAV [20]	CO,DB,R	June	7 on each treatment with washout in between	6-14	44 ATX	ADHD diagnosis, normal intelligence, minimum severity criteria	SMI, Primary sleep disorder
(Sangal;2006)		2001/October			41 MPH		
		2002			(SP II)		
LYBI [21]	DB,PC,PG,R,	Aug 2002/Sep	6	6-16	222 ATX	ADHD diagnosis, minimum severity criteria	Seizures, BPD, psychosis, PDD, concomitant psychoactive medications, anxiety, tic disorders, lack of response/tolerability issues with previous stimulant usage
(Spencer;2002)		2003			220 MPH		
					74 Pbo (SPII)		
LYBR [22]	DB, MC, R	Jan 2004/Oct	8	6-16	164 ATX	ADHD, 20-60 kg, minimum severity criteria	BPD, psychotic, PDD, suicide risk, other psychoactive medication usage, tics, tourettes,anxiety disorders
(Wang;2007)		2004			166 MPH		

*R* randomised, *DB* double-blind, *PC* placebo controlled, *MC*-multi-centre, *CO*-cross-over, *PG* parallel group, *ATX* atomoxetine, *MPH* methylphenidate, *Pbo* placebo, *ADHD* attention deficit hyperactivitydisorder, *PM* poor metaboliser, *BPD* bipolar disorder, *OBD* organic brain disease, *SMI* serious mental illness, *PDD* pervasive developmental disorder.

The FDA has defined an approach that classifies adverse events relating to suicidality in set categories [16-18]:

Code 1 = Completed suicide

Code 2 = Suicide attempt

Code 3 = Preparatory acts toward imminent suicidal behaviour

Code 4 = Suicidal ideation

Code 5 = Self-injurious behaviour, intent unknown

Code 6 = Fatal event. Not enough information

Code 7 = Self-injurious behavior, no suicidal intent

Code 8 = other: accident, psychiatric, medical

Code 9 = Not enough information (non-fatal)

Each study was searched for suicide related events using the Lilly clinical trial and serious adverse event databases as per FDA defined guidance for all events occurring during the double-blind phase or within 1 day of stopping treatment in these trials.

The following text string terms were used:

accident, asphyxiation attempt, burn, cut, drown, firearm, gas, gun, hang, hung, immolate, injure, jump, monoxide, mutilate, overdose, poison, self damage, self, harm, self inflict, self injury, shoot, slash, suffocation, suic

Patient summaries were reviewed blinded by two Eli Lilly medical staff with training and expertise in adverse event reporting and pharmacovigilance, at least one a physician. With any discrepancy a third reviewer was used. Cases were then mapped to the relevant FDA codes. Data was collected during the trial into case report forms with subsequent further additional data collected and incorporated into a patient narrative.

Meta-analytic comparisons were made using the Mantel-Haenszel risk ratio. Two variables were reported: (1) the Mantel-Haenszel risk ratio (MHRR) which estimates the percentage risk of the specific adverse event amongst ATX treated patients over the percentage among methylphenidate treated patients; and (2) the Mantel-Haenszel incidence difference (MHID) which estimates the percentage risk of the specific adverse event amongst ATX treated patients minus the percentage among methylphenidate treated patients in percentage units.

## Results

**Figure 1:**
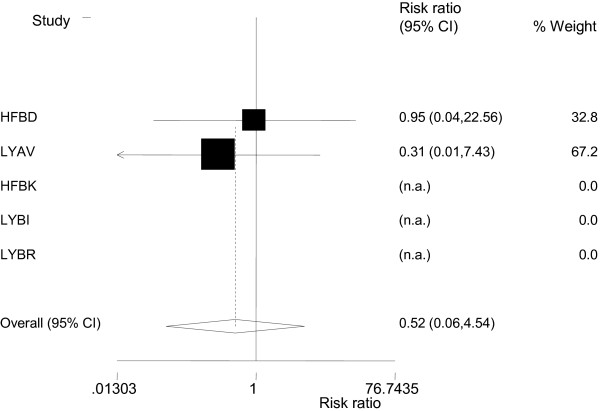
Paediatric, active comparator-controlled studies meta-analysis for FDA codes 1, 2, 3, or 4, which include all events related to either suicidal behaviour or ideation.

There are 7 comparator trials of atomoxetine and methylphenidate, 5 of which are randomised double blind and included in the analysis. Open label studies were excluded. Summaries of the characteristics of the 5 included studies, all of which were funded by Eli Lilly, are detailed in Table 1. The suicide-related events outcomes from the 5 comparator trials of atomoxetine and methylphenidate are summarised in Table 2. In total there were 5 events using FDA coding 1-9, and 2 events using FDA coding 1-4, ATX 3/559; MPH 2/465. All events using FDA coding 1-4 were suicidal ideation: there were no suicide attempts nor completed suicides. A meta-analysis on subsets of the coded adverse events and on the total coded adverse events is in Table 3 and finds no difference in risk between ATX and MPH with a Mantel-Haenszel risk ratio of 0.52 (95% CI; 0.06, 4.54) derived from the paediatric, active comparator-controlled studies for FDA codes 1, 2, 3, or 4, which include all events related to either suicidal behaviour or ideation. Individual study data are presented in Figure 1. The Mantel-Haenszel incidence difference – 0.12 (95% CI −0.62–0.38; P = 0.649) also finds no difference in rates. Brief clinical details on the individual cases are shown in Table 4.

**Table 2:** Suicide-related events: categorization of results-from acute, paediatric, active comparator-controlled studies in ADHD (FDA-defined approach)

	**Code 1**		**Code 2**		**Code 3**		**Code 4**		**Code 5**		**Code 9**	
**Study**	**ATX n/N**	**MPH n/N**	**ATX n/N**	**MPH n/N**	**ATX n/N**	**MPH n/N**	**ATX n/N**	**MPH n/N**	**ATX n/N**	**MPH n/N**	**ATX n/N**	**MPH n/N**
HFBD	0/65	0/20	0/65	0/20	0/65	0/20	1/65	0/20	0/65	0/20	0/65	0/20
HFBK	0/64	0/18	0/64	0/18	0/64	0/18	0/64	0/18	1/64	0/18	1/64	0/18
LYAV	0/44	0/41	0/44	0/41	0/44	0/41	0/44	1/41	0/44	0/41	0/44	0/41
LYBI	0/222	0/220	0/222	0/220	0/222	0/220	0/222	0/220	0/222	0/220	0/222	0/220
LYBR	0/164	0/166	0/164	0/166	0/164	0/166	0/164	0/166	0/164	0/166	0/164	1/166
TOTAL	**0/559**	**0/465**	**0/559**	**0/465**	**0/559**	**0/465**	**1/559**	**1/465**	**1/559**	**0/465**	**1/559**	**1/465**

Abbreviations: *ATX* atomoxetine, *MPH* methylphenidate.

Code 1=Completed suicide.

Code 2=Suicide attempt.

Code 3=Preparatory acts toward imminent suicidal behaviour.

Code 4=Suicidal ideation.

Code 5=Self-injurious behaviour, intent unknown.

Code 9=Not enough information (non fatal).

**Table 3:** Meta-analysis of suicide-related events in acute paediatric active comparator-controlled atomoxetine studies–ADHD (FDA-defined approach)

	**Atomoxetine**			**Methylphenidate**			**MHRR**^**a**^**(95% CI) p-value**	**MHID**^**b**^**(%) (95% CI) p-value**
**Outcome**	**No. of events**	**N**	**%**	**No. of events**	**N**	**%**		
Code 1,2,3,4: suicidal behaviour or ideation	1	559	0.18	1	465	0.22	0.52 (95% CI; 0.06, 4.54) P = 0.556	-0.12 (-0.62, 0.38) P = 0.649
Code 4: Suicidal ideation	1	559	0.18	1	465	0.22	0.52 (95% CI; 0.06, 4.54) P = 0.556	-0.12 (-0.62, 0.38) P = 0.649
Code 1,2,3,4,5,6,9: possible suicidal behaviour or ideation	3	559	0.54	2	465	0.43	0.62 (95% CI; 0.14, 2.73) P = 0.528	-0.14 (-0.88, 0.60) P = 0.713

^a^*MHRR* Mantel-Haenszel risk ratio stratified by study. It is the estimate of the percentage among atomoxetine-treated patients over the percentage among methylphenidate-treated patients.

^b^*MHID* Mantel-Haenszel incidence difference stratified by study. It is the estimate of the percentage among atomoxetine-treated patients minus the percentage among methylphenidate-treated patients in percentage units.

**Table 4:** Patients experiencing potentially suicide-related events during acute treatment (FDA-defined codes 1–6 and 9) active comparator paediatric suicidality analysis group ordered by FDA code

**Study, Inv. Patient ID**	**Brief description of event**	**Therapy**
***Paediatric ADHD, Code 4***		
HFBD	Adjustment Reaction: Suicidal Ideation	ATX
LYAV	Suicidal Ideas	MPH
***Paediatric ADHD, Code 5***		
HFBK	From Comment: took 9 capsules of med on one day	ATX
***Paediatric ADHD, Code 9***		
HFBK	Cigarette Burn to Chest	ATX
LYBR	Injury (trauma)	MPH

Abbreviations: *ATX* atomoxetine, *MPH* methylphenidate.

Code 4=Suicidal ideation.

Code 5=Self-injurious behaviour, intent unknown.

Code 9=Not enough information (non fatal).

## Conclusion

This analysis of the 5 acute randomised double-blind paediatric controlled trials of ATX and MPH of 6–9 weeks duration finds that the risk of suicide-related events as assessed using FDA methodology finds no evidence of a difference in risk between atomoxetine and methylphenidate. To our knowledge this is the only comparator data-set in existence comparing suicide-related events between these two common treatments for ADHD. [3,10,23]. Any conclusions must be regarded as tentative and hypothesis generating. Firstly due to the relatively small number of clinical trials, cohort size and 6–9 week duration. Secondly meta-analysis can have limitations particularly when analysing non-identical clinical trials. Thirdly the possibility of type 2 errors may exist in population analyses that are not specifically powered for the comparative analysis. Individually the potential risk for suicide-related adverse events is reflected by the relevant SPCs for each medication. Each drug requires monitoring for suicide-related events and the methylphenidate SPC contraindicates usage in patients with suicidal tendencies [24,25]. There are little data on suicide-related events with dexamphetamine [4].

A summary of these meta-analysis data was published in 2008 as part of an analysis of cases of suicide-related events with ATX in clinical trials [7]. Two recent cohort studies also find self harm and suicide related events are not uncommon in young people who do not have ADHD as well as those with untreated ADHD [5,6].

There are limitations however in making any finite conclusions as data derived from clinical studies are often short-term and may not be reflective of longer-term outcomes. Open-ended, rather than event-specific, adverse event solicitation may also miss suicide-related events, especially suicidal ideation. The use of the Columbia Suicide Severity Rating Scale (C-SSRS) and Columbia Classification Algorithm for Suicide Assessment (C-CASA) in clinical trials was not mandated at the time of performing these trials but is however now mandatory for ADHD medications. Suicide-related events are uncommon in a clinical trial and hence the usage of large databases to collect data over longer periods in larger cohorts may be informative [7]. Data on mortality has been reported from the United Kingdom General Practice Research Database ( UK GPRD) 1993–2006 on all patients prescribed ADHD medications [26]. Seven deaths were reported over this 13-year period and none in association with ATX. Two deaths were reported as suicide and one as “overdose of unknown intent” and all were associated with methylphenidate. The authors concluded that the standardised mortality ratio (SMR) for suicide in the 11–14 age cohort was increased but not in the 15–21 age cohort. The incident rate of suicide was 26.5/100,000 patient years in the 11–14 age cohort. No deaths of any kind were reported in the ATX cohort (n = 9,830) of a large study of two USA administrative databases of ADHD medication users compared with a control population [27]. Median follow-up however, was limited to 60 days. There have been no completed suicides in any of the clinical studies reported with atomoxetine in childhood ADHD to date and none to our knowledge in methylphenidate clinical studies.

In other defined cohorts, suicide-related events data on ATX have been reported over the last 2 years [28-30]. The importance of a control population to provide perspective is emphasised by the data from a 12- week placebo controlled study in ADHD subjects (n = 70) with comorbid substance abuse disorder (SUD) in which there were 11 cases of suicidal ideation (ATX = 4, placebo = 7) and a suicide attempt in the placebo arm [28] reported through specific questioning for these events. Suicide-related events in non-medicated ADHD subjects are well recognised and detailed history taking may show that previous suicide-related events have taken place prior to usage of ATX [5]. This facet is demonstrated well in a preliminary report of a cohort from a 1 year prescription event monitoring (PEM) study of ATX that was commenced shortly after its UK launch in 2004; in a cohort of 2544 patients, suicidal ideation 0.9%, suicide attempt 0.3%, overdose 0.3%, and 1% deliberate self harm were reported [29]. In the 23 patients reporting suicidal ideation, in the cohort of 13 patients where data was complete, 7/13 had prior history of suicidality. For the events of suicide attempt, DSH, and depression, there were 25%, 36.8%, and 25% respectively with no prior history. Thus in around three-quarters of subjects with suicide-related events there was a previous history of the same event prior to ATX usage. A high risk group may thus be defined. Longer-term data may also be helpful in defining a specific risk. In a pooled analysis of 13 placebo-controlled trials and 3 open-label extension trials in 714 atomoxetine patients treated for more than 3 years, there were 1.5% patients with suicidal ideation, 0.3% suicide attempts, and 0.1% suicidal behaviours, involving 14/714 patients [30]. In the absence of a control population it is difficult to put these data into perspective.

Suicide-related events may be associated with the ingestion of ADHD medications [23,24]. The usage of the FDA defined C-CASA coding for suicidality and the incorporation of the C-SSRS into future clinical studies may also provide important data regarding suicide-related events in ADHD treated cohorts [17].

This meta-analysis and current data support that suicide-related events are measurable in an ADHD treated cohort but that there is no current evidence of any significant differential risk between ATX and MPH, however this is the only systematic evidence currently available on suicide-related events in patients receiving psychostimulants and data from ongoing clinical trials may be helpful in defining further this comparison [23]. It is also salient to note that our data derive from a clinical trial cohort of patients who may not be representative of patients in the real world. Patients with certain comorbid illnesses were excluded from the trials yet may present for ADHD treatment to clinicians. There is thus no certainty that these data would be reflective of a non clinical trial cohort. One study excluded entrants deemed to be at risk of suicide and another study those with known stimulant tolerability issues. Further limitations of such studies that are not specifically designed to study suicide-related events include the potential for underreporting which may also lead to type 2 errors.

Clinicians have no current reason to solely choose their treatments on the basis of any presumption of differential risk of suicide-related events. The usage of Columbia Suicide Severity Rating Scale (C-SSRS) and Columbia Classification Algorithm for Suicide Assessment (C-CASA) in clinical trials where appropriate, when used prospectively may further help to define any suicidal risk in clinical trials [17]. Longer-term studies in larger populations such as ADDUCE (Attention deficit/hyperactivity disorder drugs use chronic effects), will further define risk of suicide-related events in the treated populations outside of clinical trials designed for drug registration [31].

The risk of suicide associated with ADHD must not be underestimated. A recent birth cohort study reported that 1.9% of an ADHD cohort (mean age diagnosis 10 years) were deceased (all causes including suicide) at follow-up (mean age 27 years), with the standardised mortality ratio for suicide as an individual cause of death elevated 4.83 (95% CI 1.14–20.46) when compared with a control cohort [32]. A recent editorial also confirms the view that ADHD is associated with elevated risk for not only suicide related behaviours but also suicide and advises screening for suicide attempts even in younger populations with ADHD [33]. Fortunately each SPC for ADHD medication provides clear guidance on contraindications, warnings and monitoring, with the aim of reduction in all suicide related events.

## Competing interests

CB and NS are employees and stockholders of Eli Lilly and Company who manufacture atomoxetine.

## Authors’ contributions

CB and NS conceived the project. The first draft was written by CB and subsequent revisions by CB and NS. Both authors read and approved the final manuscript.
